# Incidence of household catastrophic and impoverishing health expenditures among patients with Breast Cancer in Iran

**DOI:** 10.1186/s12913-021-06330-6

**Published:** 2021-04-09

**Authors:** Faranak Ahmadi, Hamidreza Farrokh-Eslamlou, Hasan Yusefzadeh, Cyrus Alinia

**Affiliations:** 1 Department of Health Management and Economics, School of Public Health, Urmia University of Medical Sciences, Urmia, Iran; 2 Reproductive Health Research Centre, School of Public Health, Clinical Research Institute, Urmia University of Medical Sciences, Urmia, Iran

**Keywords:** Iran, Breast cancer, Catastrophic health expenditures, Impoverishing health expenditures, Out-of-pocket

## Abstract

**Background:**

Breast cancer disease is the most common cancer among Iranian women and imposing a significant financial burden on the households. This study calculated out-of-pocket (OOP), catastrophic health expenditure (CHE), and impoverishing health spending attributed to breast cancer in Iran.

**Methods:**

In this cross-sectional household study, clinical and financial information on breast cancer and also household information (expenditures and income) were obtained through face-to-face interviews and completing a questionnaire by 138 women with this disease in 2019. We applied three non-food expenditure thresholds of 40, 20, and 10% to defining the CHE. Disease costs included periodical visits, diagnostic services, hospitalization care, treatment and rehabilitation services, home, and informal care. Households were disaggregated into socioeconomic status quintiles based on their Adult Equivalent values standardized monthly consumption expenditures. To identify the factors affecting these indicators, we performed the two different multivariate logistic regression models.

**Results:**

This study finds that each patient had a monthly average OOP payment of $US 97.87 for the requested services, leading to impoverished of 5.07% and exposed 13.77% of their households to CHE. These indicators have been mainly concentrated among the poor, as they have spent a large part of their meager income on buying the needed services, and for this purpose, most of them forced to sell their assets, borrow, or take a bank loan.

**Conclusions:**

The patients in lower SES quintiles can be protected from impoverishing and catastrophic health spending by expanding insurance coverage, providing financial risk protection programs, and increasing access to quality and effective public sector services. Alongside, expanding inpatient coverage and adding drug benefits for the poor can significantly decrease their OOP payments.

## Background

Breast cancer is the most common cancer and is the second leading cause of cancer death among Iranian women and the western world [1]. Annually, more than 1.2 million patients are diagnosed with this age-related disease, of which more than 500,000 dies [2]. In other words, one out of every 8 to 10 women in the world and 10 to 15 women in Iran get breast cancer [3]. The prevalence of breast cancer in Iran has increased dramatically over the past two decades due to lifestyle changes [4]. Iranian women get the disease on average a decade earlier than their Western counterparts [5].

Breast cancer is a chronic disease that requires expensive diagnostic, treatment, and rehabilitation services. In the long run, this disease imposes high costs on the patients, families, and health systems [6–8]. The disease management requires frequent hospitalization courses, receiving specialized laboratory tests, surgery, chemotherapy, radiotherapy, expensive drugs, outpatient visits, and continuous follow-up [9]. The disease, especially in more advanced stages, leads to unemployment and reduced income, implying additional economic pressure on the patient and her family [6–8]. This financial pressure is even more tremendous for vulnerable groups such as poor or unemployed households [6].

The average direct cost of breast cancer treatments varies from $US 222.17 to $US 828.52, depending on the disease’s stage and progression [10]. The condition imposes $US 947,374,468 annually on involving Iranian households, that about 77% of which is pertained to productivity lost. Also, chemotherapy accounts for the largest share of the disease’s direct costs, at about $US 77 million per year [11].

The most effective approach to controlling mortality, disease burden, and breast cancer costs is early detection programs that use mammography as the screening test, which has been pursued in developed countries over the past decade and has achieved considerable successes [12, 13]. Of course, this screening approach is not cost-effective in low and middle-income countries such as Iran [14–16]. Given that the disease is diagnosed in advanced stages in developing countries, it has left more costs and twice as many deaths as in developed countries [16].

The Iranian health system implemented some policies to reduce out-of-pocket payments to a maximum of 6% of total costs in the public sector, increasing insurance coverage, and regulating the market to cushion breast cancer patients against the costs [17]. Due to the lack of insurance coverage for some needed services and medicines and receiving part of the benefits from the private sector, it seems that the health system has not had much success in this field [18].

Although minimal information and documentation are available on the cost of breast cancer management, given the above, this hypothesis arises that households struggling with the disease significantly exposed to catastrophic and impoverishing health expenditures. In this study, we have tried to test this hypothesis and fill this knowledge gap in Iran.

If a household is forced to lessen their living subsistence over a while due to OOP fees for health care services, the payments are catastrophic. Depending on the definition of basic needs used to calculate household capacity to pay for health care, we have four CHE calculating methods. The simplest way, the budget share method, does not define basic needs and considers all household expenditures as capacity to pay. This method is used to monitor universal health coverage in the sustainable development goals. But in the two approaches of actual food spending and partial normative food spending, to calculate the household capacity to pay, respectively, household actual food spending and average food spending per (equivalent) a person are defined as living subsistence. In the fourth method, normative spending on food, housing, and utilities, the basic needs are obtained as the sum of food, rent, and utilities spent per (equivalent) a person. All the approaches select OOP payments as the numerator for calculating the incidence of CHE [19, 20].

## Methods

### Study design and data collection

In this cross-sectional study, breast cancer information was obtained through face-to-face interviews and completing a questionnaire by 138 women with this disease in Urmia, Iran, in 2019. This information covered the patients and heads of the household’s demographic data, household expenses and income, clinical data on the disease, and funding sources for the disease management. We randomly selected the samples from a list of registered patients in the private and public specialized centers of Urmia University of Medical Sciences. To minimize the number of missed cases, at least three times at two-week intervals, we contacted any respondent who was not ready for the interview for any reason and conducted an interview with her. The inclusion criteria were having at least 25 years of age, passing at least 1 month since the definitive diagnosis of the disease, and been living in Iran in the past year.

### Measuring catastrophic breast cancer expenditures

Technically, health expenditure is considered catastrophic when OOP health spending exceeds a specific ratio of household capacity to pay. This threshold is set by the World Health Organization (WHO) at 40%, but other standards, such as 20 and 10%, have been introduced in various related studies. Household capacity to pay is obtained by subtracting the average monthly cost of subsistence from the average monthly effective income (total consumption expenditure) of the household over the past year. In other words, the capacity to pay is defined as non-subsistence expenses. Based on the WHO recommendation, we consider subsistence expenses to be equal to household food expenditures [21]. Food expenses make up a significant fraction of household expenses, have a low-income elasticity, and are strongly influenced by household members [18]. Household food expenditures include the household’s costs in purchasing foodstuffs and the financial value of the food products produced and consumed by the household itself. We excluded the food expenses incurred by households in restaurants and hotels from these calculations. The expenditure variable can better show the household purchasing power than the income variable, especially in developing countries [22]. Given that household expenditures can have an unbalanced distribution during different months of the year, we asked the participants about their average monthly household expenditure over the past year, which included all of the following items; food, beverages, recreation, education services, hotel and restaurant, clothing and footwear, cigarettes and tobacco, house and shop rent, housing, water, fuels (gas, electricity, and other possible fuels), transportation، communication، household appliances، furniture، health, and financial value of any consumption of household products (agricultural, services, industrial, etc.).

Considering the diminishing marginal utility of remaining household consumption expenditure after subtracting the Out-of-pocket (OOP) health payment from effective income for different SES quintiles, we used three levels of non-food expenditure thresholds; 10, 20, and 40%. OOP payments refer to the spending made by households at the point they receive breast cancer services, which cover the periodical visits, diagnostic services, hospitalization care, treatment services (drugs, chemotherapy, radiotherapy, surgery, and others), rehabilitation services, home cares provided by the physician, nurse, and household members, and finally receive informal care from traditional therapists.

### Measuring impoverishing due to breast cancer spending

The impoverishing payment is measured by the proportion of households that falls below the absolute poverty line after breast cancer spending is subtracted from total household consumption [23]. Such households can still not meet their basic food needs, even if they spend all their remaining expenses on them. The exchange rate used for conversion is 1 $US = 135,000 Iranian Rials. According to a report by the Iranian Ministry of Labor, the monthly cost of a subsistence basket for each person was 11,393,940 Rials (84.40 PPP $US) in 2019, considered the poverty line [24]. Therefore, by multiplying this value by the standardized household dimension, each household’s absolute poverty line is calculated. If the total household expenditure was less than their equivalent absolute poverty line, that household was considered poor. If the total remaining household expenditure goes below this estimated line due to the cost of treating breast cancer, that household will incur impoverishing spending. Also, we asked the participants the sources of funding for the disease.

### Statistical analysis

As we used the partial normative food spending approach, households were disaggregated into SES quintiles based on their monthly consumption expenditures, where SES classifications were standardized by Adult Equivalent (AE) values of consumption expenditures. For this, we applied the formula presented by Cirto and Michael [25] as follow:

$$ AE={\left(A+\alpha K\right)}^{\theta } $$

Where A = number of adults (aged over 18 years), K = number of children, α = cost coefficient for children, and θ = degree of economies of scale. The recommended values for α and θ for developing countries are 0.4 and 1.0, respectively [25].

For statistical comparisons of the incidence of catastrophic and impoverishing spending on breast cancer treatment between different sociodemographic subgroups, we applied the independent t-test and one-way ANOVA respectively for two and more than two separate subgroups. In this analysis, the *p*-values which are equal to or below 0.05 are considered to be significant.

To identify the factors affecting catastrophic and impoverishing health expenditures for breast cancer treatment, we performed the two separate multivariate logistic regression models with the following dependent variables:

- Disease characteristics (cancer type, disease duration, treatment types).
- Patient characteristics (age, educational level, place of living, marital status, insurance status, household size).
- Head of household characteristics (age, educational level, marital status).

Those variables that had multicollinearity problems were omitted from the models. We estimated and reported the odds ratio, 95% confidence interval (CI), and *p*-value statistics for each variable. One-Sample Kolmogorov–Smirnov test checked the normality distribution of the variables. All statistical analyses were performed by STATA version 15.0 software (Stata Corp, College Station, TX).

## Results

The findings indicate that the mean age of 138 patients participating in the study was 44.3 years (SD: 11.73), about a third of whom were illiterate and often lived in the urban. Also, more than 97% of them have at least one type of basic health insurance (Table 1).

**Table 1:** Characteristics of the study sample

Variables	Head of household	Patient
	*N* = 138 Frequency (%)	
Gender (female)	19 (13.77)	138 (100)
Mean of age (SD)	65.32 (13.40)	44.30 (11.73)
Education level		
Illiterate	34 (24.64)	45 (32.61)
Elementary	31 (22.46)	33 (23.91)
Middle school	10 (7.25)	14 (10.14)
Higher school	40 (28.99)	35 (25.36)
University	23 (16.67)	11 (7.97)
Marital status		
Single	4 (2.90)	14 (10.14)
Married	120 (86.96)	108 (78.26)
Divorced/Widowed	14 (10.14)	16 (11.60)
Having basic health insurance		
Yes	134 (97.10)	134 (97.10)
No	4 (2.90)	4 (2.90)
Having complementary insurance		
Yes	37 (26.81)	36 (26.09)
No	101 (73.19)	102 (73.91)
Mean of household dimension (SD)	3.89 (0.12)	
Place of living		
Urban	84 (60.87)	
Rural	54 (39.13)	

Table 2 provides findings on amounts of out-of-pocket payment and the incidence of impoverishing and catastrophic breast cancer management expenditures in different SES quintiles. The results show that Iranian households’ average amount to receive diagnostic, treatment, and rehabilitation services for breast cancer was $US 97.87 annually. This amount was obtained for the poorest and richest quintiles at $US 69.97 and 140.83, respectively, which their difference was statistically significant. The findings also show that, with a threshold of 40% of the household’s capacity to pay, an average of 13.77% of the households have suffered from catastrophic health expenditure (CHE) caused by breast cancer. This incidence value is estimated at 27.54 and 40.58%, respectively, for 20 and 10% of non-food expenditure. In this regard, there was no statistically significant difference between SES quintiles in all three models. However, the proportion of households incurring CHE in the first to the fifth quintile was between 1.10–1.61.

**Table 2:** Brest cancer out-of-pocket, catastrophic, and impoverishment health expenditures by SES quintiles (Monthly data-$US)

Expenditure indexes		Percent of households with CHE at different threshold			OOP expenditures (SD)	Percent of impoverished households
		40%	20%	10%		
SES Quintiles	Q1 (poorest)	22.22	33.33	44.44	69.97 (66.95)	18.52
	Q2	11.11	18.52	29.63	68.70 (53.51)	0.00
	Q3	11.11	25.93	33.33	82.33 (63.18)	3.70
	Q4	14.29	28.57	53.57	128.22 (119.52)	3.57
	Q5 (richest)	10.34	31.03	41.38	140.83 (98.94)	0.00
	All households	13.77	27.54	40.58	97.87 (89.30)	5.07
	Q1:Q5 ratio	1.61	1.21	1.10	0.50	3.65^b^
	Chi square for trend (P-value)	2.24 (0.69)	1.78 (0.78)	4.07 (0.40)	4.81 (0.001)^a^	13.37 (0.01)

^a^One-way ANOVA test is used

^b^is the result of first quintile by the average value

The results also show that the cost of managing breast cancer disease causes 18.52% of households belonging to the first quintile to become poor, compared to zero in households belonging to the fifth quintile. The incidence average of impoverishing expenditure was estimated at 5.07.

Table 3 depicts the incidence of catastrophic and impoverishing payments caused by breast cancer disease in different socio-economic subgroups. The CHE for benign patients, patients treated with radiotherapy, patients who have lived in rural areas, patients belong to the age group of 40–65, illiterate, and single patients were higher than others. However, this difference was statistically significant only for the type of treatment variable. The incidence of impoverished households pertained to breast cancer costs ranged from zero (for older subgroups, undergraduate, undergoing surgery, and patients without health insurance) to 9.52% (for radiotherapy treatment) in different socio-demographic subgroups. Of course, there was no statistically significant difference between the subgroups of each socio-demographic group.

**Table 3:** Household catastrophic health expenditures and impoverished costs of breast cancer among different socio-demographic groups of the patients

SES subgroups		Number (%)	Percent of households with CHE at different thresholds			Percent of impoverished households
			40%	20%	10%	
Cancer type	Benign	61 (44.20)	18.03	32.79	42.62	4.92
	Malignant	77 (55.80)	10.39	23.38	38.96	5.19
Disease duration	< 1 years	117 (74.78)	13.68	28.21	41.88	5.13
	> 1 years	21 (25.22)	14.29	23.81	33.33	4.76
Treatment type^a b^	Chemotherapy	61 (44.20)	8.20	26.23	37.70	3.28
	Radiotherapy	5 (3.62)	60.00	100.00	100.00	9.52
	Surgery	42 (30.43)	23.81	35.71	40.48	0.00
	Others	30 (21.74)	3.33	6.67	36.67	3.33
Place of living	Urban	84 (60.87)	9.52	23.81	40.48	4.76
	Rural	54 (39.13)	20.37	33.33	40.74	5.56
Household dimension	< 5 persons	96 (69.57)	14.58	29.17	40.63	4.17
	> 4 persons	42 (30.43)	11.90	23.81	40.48	7.14
Age	< 40 years	31 (23.31)	9.68	16.13	29.03	3.23
	40–65 years	95 (71.43)	14.74	30.53	44.21	5.26
	> 65 years	7 (5.26)	0.00	14.29	28.57	0.00
Having insurance	Yes	134 (97.10)	14.18	28.36	41.04	5.22
	No	4 (2.90)	0.00	0.00	25.00	0.00
Education level	Illiterate	45 (32.61)	20.00	33.33	44.44	8.89
	< University	82 (52.49)	12.20	24.39	36.59	3.66
	University	11 (7.97)	0.00	27.27	54.55	0.00
Marital status	Married	108 (78.26)	12.04	25.93	37.96	5.56
	Unmarried	30 (21.74)	20.00	33.33	50.00	3.33

^a^indicates significant difference for catastrophic health expenditures at 40% threshold between subgroups

^b^indicates significant difference for catastrophic health expenditures at 20% threshold between subgroups

Table 4 presents our regression results. As the results suggest, if we consider the 40% as a threshold level, odds of CHE statistically significantly was higher among respondents who have used the radiotherapy (odds ratio: 1.33, 95% CI: 1.14–1.52) and were lower for elderly patients (odds ratio: 0.28, 95% CI: 0.14–0.62) and the patients with university education (odds ratio: 0.27, 95% CI: 0.21–0.33) compared with their reference subgroups. These results are not confirmed in another approach, which considers 10% as the threshold level. Besides, the findings did not show any statistically significant association between impoverishing breast cancer payments with dependent factors except for the patients who had a university degree (odds ratio: 0.37, 95% CI: 0.12–0.66).

**Table 4:** Determinants of catastrophic and impoverishing health expenditure and: a multivariate logistic regression model

Variables	Reference subgroup	CHE at different thresholds						Impoverishing payments		
		≥ 40% of CTP			≥ 10% of CTP					
		OR	95% CI	p	OR	95% CI	p	OR	95% CI	p
Disease characteristics										
Cancer type	Benign	0.64	0.24–1.04	0.12	0.64	0.39–1.31	0.27	0.76	0.13–2.63	0.77
Disease duration	< 1 years	0.68	0.23–1.13	0.60	0.58	0.29–1.55	0.34	0.95	0.48–2.13	0.87
Treatment types	Chemotherapy									
Radiotherapy		1.33	1.14–1.52	0.01	1.26	0.04–1.51	0.16	1.31	0.60–2.84	0.22
Surgery		1.24	0.94–1.55	0.13	1.12	0.85–1.39	0.25	1.18	0.47–2.36	0.47
Patient characteristics										
Age groups	< 40 years									
40–65 years		1.81	1.30–3.32	0.04	3.59	1.03–9.54	0.04	1.32	0.71–3.74	0.48
> 65 years		0.28	0.14–0.62	0.01	0.62	0.25–1.15	0.19	0.54	0.28–2.31	0.21
Educational level	Illiterate									
< University		0.80	0.55–1.05	0.61	1.06	0.45–2.52	0.90	1.17	0.52–3.61	0.89
University		0.27	0.21–0.33	0.01	1.21	0.58–2.46	0.77	0.37	0.12–0.66	0.01
Place of living	Urban	2.66	1.45–3.88	0.17	0.75	0.29–1.93	0.56	0.86	0.10–3.15	0.90
Marital status	Married	1.36	0.78–1.94	0.80	0.36	0.10–1.23	0.10	–	–	–
Household size	< 5 persons	0.59	0.15–2.28	0.45	0.75	0.32–1.80	0.53	0.89	0.13–2.15	0.72
Have insurance	No	0.66	0.28–1.04	0.18	0.22	0.09–0.43	0.02	–	–	–
Head of household characteristics										
Age groups	< 50 years	0.97	0.89–1.04	0.36	0.96	0.91–1.00	0.07	1.07	0.94–1.24	0.28
Educational level	Illiterate									
< University		0.94	0.57–1.55	0.81	0.90	0.64–1.26	0.53	0.63	0.27–1.53	0.32
University		0.79	0.45–1.11	0.55	0.77	0.48–1.06	0.48	0.79	0.31–1.17	0.09
Marital status	Married	2.14	0.54–8.54	0.28	0.56	0.25–1.26	0.16	–	–	–

Variables of marital statuses and health insurance condition of the patients are omitted due to collinearity problem in improvising payment model

## Discussion

This cross-sectional study was investigated the OOP payments, catastrophic, and impoverishing health spending attributed to breast cancer in Iran. The results indicate that each household with a breast cancer patient pays a monthly average of $US 97.87 for diagnostic, treatment, and rehabilitation services. It is much lower than the estimated OOP for similar patients in Ontario ($US 393) [26], the rural of Florida ($US 253.2) [6], and Nepal ($US 289.78) [27]. However, our results strongly support the findings of these similar studies that there is a statistically significant difference among SES quintiles in the OOP payments. As the higher quintiles, on average, have higher OOP payments to receiving the services [6, 26, 27].

### CHE and breast cancer

Depending on threshold levels of 40, 20, and 10% of the household’s capacity to pay, the CHE’s incidences caused by breast cancer are estimated as 13.77, 27.54, and 40.58%. It means that in the best-case scenario, 13.7% of the Iranian households involved with the disease suffered from CHE and had forced to sacrifices the consumption of other goods and services necessary for their well-being. This is much lower than estimates made by other comparable studies in Iran (60.9%) [28], China (30.98%) [29], Ethiopia (72.3%) [30], Vietnam (71.8%) [31], Haiti (67%) [32], and South Korea (39.8%) [33]. Although households with higher SES levels are less likely to incur CHE, a significant fraction of all SES subgroups are exposed to these limiting costs. This amount is estimated between 10.34 to 41.38% for the most affluent segment of society in different scenarios that was more than our expectation, as these subgroups spent a smaller fraction of their budget on the required health care services than others. This unexpected finding could be because the higher SES subgroups receive most of the needed services from the private sector, which their tariffs are far higher than the cost of similar services in the public sector.

It is interesting to note that those 40–65 years old were 1.81 times more likely, and the patients older than 65 were 3.57 times less likely to experience CHE than the younger subgroup. This significant difference could be because of their source of services and illness duration. The elderly and middle-aged patients mostly use the cheaper care of public hospitals and expensive care of private clinics, respectively. Besides, unlike middle-aged people, the elderly have shorter illness duration because of their older age and high mortality rate and incur lower costs. This study also discovered a significant negative association between education level and CHEs’ incidence, as those with a university degree were 3.7 times less likely to experience CHE than illiterate people. One reason that could explain this finding is that people with higher education levels usually have more healthy living conditions and receive faster and more effective preventive care. Also, education level has a significant and positive association with SES level, as people with higher education levels have more income than others. Thus, even with the same fees for receiving the required services, this group is less likely to be exposed to catastrophic and impoverishing payments [6]. Besides, the patients who received radiotherapy treatment were more likely to incur CHE compared with others. These findings support evidence from previous investigations [33–36].

### Impoverishing expenditure and breast cancer

CHE’s high incidence has significantly increased the risk of household impoverishment, with 5.07% of the households being impoverished due to OOP breast cancer payments. Of course, most of these households belonged to the lower sections of society, so that the incidence of impoverishing health spending in the lowest quintile was equal to 18.52% and in the wealthiest quintile was equal to zero. This investigation’s findings were higher than those of other studies conducted among nationally representative households in Iran, China, and Vietnam [37–39] and lower than cancer patient households of Vietnam [31]. Impoverishment from financing breast cancer care has only had a significant negative relationship with education level. Patients with a university education level were about 2.7 times less likely to become poor than illiterate patients. Further analysis of the data confirmed that most patients with university education level belonged to the high-income subgroup. Thus, the education level was only a confounding variable, and this observed relationship was mainly due to the high level of household income.

### Study limitations

The findings must be interpreted in light of this limitation that, as other research on household data, this study also relied on self-reported costs and incomes, subject to recall bias. Thus, it is not excluded that patients forgot, underestimated, or overestimated some of the asked information. Besides, we used three fixed threshold levels to consider the diminishing marginal utility of capacity to pay across the different SES quintiles. Nevertheless, since the level of non-food expenditures that leads to catastrophic costs in different SES quintiles is not the same, it seems that we underestimate the breast cancer’s CHE [40]. The solution suggested by Onoka et al. [18] is to use the variable threshold level method. In that approach, the levels for various SES groups were weighted by the ratio of household expenditure on food. Therefore, we recommend that the variable threshold level method be used in future studies to calculate the CHE of Breast cancer more accurately.

### Policy implications

Identifying the at-risk subgroups for catastrophic and impoverishing payments and identifying these risk sources can be critical in effective policy-making to reduce these indicators. The findings show a greater likelihood of catastrophic expenses among those who require radiotherapy treatment, are in their middle-age, and are in the lower SES class, which recommend the policymakers this population has the greatest need to develop risk pooling health financing approaches. Also, heavy reliance on OOP financing leaves the households exposed to the risk of unforeseen medical expenditures. Then, any considered solution should aim to reduce OOP payments to decrease the risk of CHE and Impoverishing costs incidence.

Therefore, it is recommended that the benefits package of primary services for patients with breast cancer, namely chemotherapy, radiotherapy, surgery, and high-cost drugs for at-risk households, be covered by health insurance to relieve the disease’s financial burden. Besides, government supporting programs and providing access to quality services in public health centers for these patients can significantly reduce the OOP payments.

Another key to solving the problem is knowing the source of payment for treating the disease among these households. The study’s findings showed that payments were made from various sources, of which current household income and health insurance were the primary sources of payment, but they were not sufficient. More than two-thirds of the households used their savings to finance their medical expenses, and more than a third of them sold their capital goods. Also, about a quarter and 16% of the households were forced to borrow and take a bank loan, respectively. (Table 5) This finding broadly supports the work of other studies in this area in Iran [28, 41], Ethiopia [30, 42], India [43], and Australia [44].

**Table 5:** Sources of payment for breast cancer costs among the participants

Sources	Current income	Health insurance	Saving	Capital^a^ sales	Borrowing	Bank loans
Number	134	134	93	51	34	22
Percent	97.10	97.10	67.39	36.96	24.64	15.94

^a^Such as stock, gold, home appliances, and other similar cases

## Conclusions

In this cross-sectional investigation, we applied a retrospective reporting of household costs on breast cancer disease management to calculating the OOP, impoverishing, and catastrophic breast cancer expenditure. This study finds that each patient had a monthly average OOP payment of $US 97.87 for the requested services, leading to impoverished of 5.07% and exposed to CHE of 13.77% of their households. These indicators have been mainly concentrated among the poor, as they have spent a large part of their meager income on buying the needed services, and for this purpose, most of them have been forced to sell their assets, borrow, or take a bank loan. Therefore, the patients in lower SES quintiles can be protected from CHE and impoverishing health spending by expanding insurance coverage, providing financial risk protection programs, and increasing access to quality and effective public sector services. Alongside, expanding inpatient coverage and adding drug benefits for poor can significantly decrease their OOP payments.

## Data Availability

The datasets generated and/or analyzed during the current study are not publicly available for confidentiality reasons since individual privacy could be compromised but are available from the corresponding author on reasonable request.

## Abbreviations

OOP: Out-of-pocket
CHE: Catastrophic health expenditure
SES: Socio-economic status
WHO: World Health Organization
SD: Standard deviation
CI: Confidence Interval

## References

[1] Nafissi N, Khayamzadeh M, Zeinali Z, Pazooki D, Hosseini M, Akbari ME (2018). Epidemiology and histopathology of breast cancer in Iran versus other middle eastern countries. Middle East J Cancer.

[2] AsghariEbrahimAbad MJ, Kareshki H (2019). Psychological consequences of breast Cancer in Iran: a systematic review. J Fasa Univ Med Sci.

[3] Kazemzadeh S, Babaei E (2018). Investigating the expression of CCAT2 gene as a new molecular marker in breast tumors. J Fasa Univ Med Sci.

[4] Tahergorabi Z, Moodi M, Mesbahzadeh B (2014). Breast Cancer: a preventable disease. J Birjand Univ Med Sci.

[5] Abolghasemi J, Asadi Lari M, Mohammadi M, Salehi M (2013). Effective factors in the appearance of metastasis in patients with breast cancer using frailty model. J Arak Univ Med Sci.

[6] Pisu M, Azuero A, Benz R, McNees P, Meneses K (2017). Out-of-pocket costs and burden among rural breast cancer survivors. Cancer Med.

[7] Pisu M, Azuero A, McNees P, Burkhardt J, Benz R, Meneses K (2010). The out of pocket cost of breast cancer survivors: a review. J Cancer Surviv.

[8] Mols F, Tomalin B, Pearce A, Kaambwa B, Koczwara B: Financial toxicity and employment status in cancer survivors. A systematic literature review. Support Care Cancer 2020;28(12):1–16. 10.1007/s00520-020-05719-z PMC7686183 32865673

[9] Bazyar M, Pourreza A, Harirchi I, Akbari F, Mahmoudi M (2012). Medical and non-medical direct costs of cancers in patients hospitalized in imam Khomeini cancer institution-2010. Hospital.

[10] Davari M, Yazdanpanah F, Aslani A, Hosseini M, Nazari AR, Mokarian F (2013). The direct medical costs of breast cancer in Iran: analyzing the patient's level data from a cancer specific hospital in Isfahan. Int J Prev Med.

[11] Daroudi R, Sari AA, Nahvijou A, Kalaghchi B, Najafi M, Zendehdel K (2015). The economic burden of breast cancer in Iran. Iran J Public Health.

[12] Advani S (2004). Partner profile: cancer in India. INCTR News.

[13] Ferlay J, Bray F, Pisani P, Parkin D: Cancer incidence, mortality and prevalence worldwide, version 1.0. IARC Cancer base 2001;5:2004.

[14] Barfar E, Rashidian A, Hosseini H, Nosratnejad S, Barooti E, Zendehdel K (2014). Cost-effectiveness of mammography screening for breast cancer in a low socioeconomic group of Iranian women. Arch Iran Med.

[15] Haghighat S, Akbari ME, Yavari P, Javanbakht M, Ghaffari S: Cost-effectiveness of three rounds of mammography breast cancer screening in Iranian women. Iran J Cancer Prevent 2016;9(1):5443. 10.17795/ijcp-5443 PMC4922208 27366315

[16] Okonkwo QL, Draisma G, der Kinderen A, Brown ML, de Koning HJ (2008). Breast cancer screening policies in developing countries: a cost-effectiveness analysis for India. JNCI.

[17] Afkar A, Heydari S, Jalilian H, Pourreza A, Sigaroudi AE (2020). Hospitalization costs of breast cancer before and after the implementation of the health sector evolution plan (HSEP), Iran, 2017: a retrospective single-Centre study. J Cancer Policy.

[18] Onoka CA, Onwujekwe OE, Hanson K, Uzochukwu BS (2011). Examining catastrophic health expenditures at variable thresholds using household consumption expenditure diaries. Tropical Med Int Health.

[19] Thomson S, Cylus J, Evetovits T (2019). Can people afford to pay for health care? New evidence on financial protection in Europe. Eurohealth.

[20] Cylus J, Thomson S, Evetovits T (2018). Catastrophic health spending in Europe: equity and policy implications of different calculation methods. Bull World Health Organ.

[21] Mehrdad R (2009). Health system in Iran. JMAJ.

[22] O'donnell O, Van Doorslaer E, Wagstaff A, Lindelow M: Analyzing health equity using household survey data: a guide to techniques and their implementation: The World Bank; 2007, doi: 10.1596/978-0-8213-6933-3.

[23] Wagstaff A: Measuring financial protection in health: The World Bank; 2008, doi: 10.1596/1813-9450-4554.

[24] Shahbazian A, Abdollahi M, Einian M, Kaviani Z: Estimating the poverty line for the first six months of 1397. Tehran: Parliament Research Centre of IRI 2018.

[25] Council NR: Measuring poverty: a new approach. Tehran: National Academies Press; 1995.

[26] Longo CJ, Bereza B: A comparative analysis of monthly out-of-pocket costs for patients with breast cancer as compared with other common cancers in Ontario, Canada Curr Oncol 2011;18(1):681, e8, doi: 10.3747/co.v18i1.681. 10.3747/co.v18i1.681 PMC3031360 21331267

[27] Khatiwoda SR, Dhungana RR, Sapkota VP, Singh S (2019). Estimating the direct cost of cancer in Nepal: a cross-sectional study in a tertiary cancer hospital. Front Public Health.

[28] Kavosi Z, Delavari H, Keshtkaran A, Setoudehzadeh F (2014). Catastrophic health expenditures and coping strategies in households with cancer patients in shiraz Namazi hospital. Middle East J Cancer.

[29] Zheng A, Duan W, Zhang L, Bao X, Mao X, Luo Z, Jin F (2018). How great is current curative expenditure and catastrophic health expenditure among patients with cancer in China? A research based on “system of health account 2011”. Cancer Med.

[30] Kasahun GG, Gebretekle GB, Hailemichael Y, Woldemariam AA, Fenta TG (2020). Catastrophic healthcare expenditure and coping strategies among patients attending cancer treatment services in Addis Ababa, Ethiopia. BMC Public Health.

[31] Hoang VM, Pham CP, Vu QM, Ngo TT, Tran DH, Bui D, Pham XD, Tran DK, Mai TK: Household financial burden and poverty impacts of cancer treatment in Vietnam BioMed Res int. 2017;2017:1-8. 10.1155/2017/9350147 PMC5585616 28904976

[32] O'Neill KM, Mandigo M, Pyda J, Nazaire Y, Greenberg SL, Gillies R, Damuse R (2015). Out-of-pocket expenses incurred by patients obtaining free breast cancer care in Haiti: a pilot study. Surgery.

[33] Choi J-W, Cho K-H, Choi Y, Han K-T, Kwon J-A, Park E-C (2014). Changes in economic status of households associated with catastrophic health expenditures for cancer in South Korea. Asian Pac J Cancer Prev.

[34] Su TT, Kouyaté B, Flessa S (2006). Catastrophic household expenditure for health care in a low-income society: a study from Nouna District, Burkina Faso. Bull World Health Organ.

[35] Song E-C, Shin Y-J (2010). The effect of catastrophic health expenditure on the transition to poverty and the persistence of poverty in South Korea. J Prev Med Public Health.

[36] Kavosi Z, Rashidian A, Pourreza A, Majdzadeh R, Pourmalek F, Hosseinpour AR, Mohammad K, Arab M (2012). Inequality in household catastrophic health care expenditure in a low-income society of Iran. Health Policy Plan.

[37] Rezapour A, Ghaderi H, Azar F, Larijani B, Gohari MR (2013). Effects of health out-of-pocket payment on households in Iran; catastrophic and impoverishment: population based study in Tehran (2012). Life Sci J.

[38] Zhao Y, Oldenburg B, Mahal A, Lin Y, Tang S, Liu X (2020). Trends and socio-economic disparities in catastrophic health expenditure and health impoverishment in China: 2010 to 2016. Tropical Med Int Health.

[39] Van Minh H, Phuong NTK, Saksena P, James CD, Xu K (2013). Financial burden of household out-of pocket health expenditure in Viet Nam: findings from the National Living Standard Survey 2002–2010. Soc Sci Med.

[40] MANAVGAT G, SAYGILI F, AUDIBERT M (2020). Examining the economic burden of out-of-pocket health expenditures for households in different socio-economic groups in Turkey. Sosyoekonomi.

[41] Pourreza A, Harirchi I, Bazyar M (2017). Differentiation of out-of-pocket expenditures in cancer patients; a case study in the cancer institute of Iran. Evid Based Health Policy Manage Econ.

[42] Tolla MT, Norheim OF, Verguet S, Bekele A, Amenu K, Abdisa SG, Johansson KA: Out-of-pocket expenditures for prevention and treatment of cardiovascular disease in general and specialised cardiac hospitals in Addis Ababa, Ethiopia: a cross-sectional cohort study. BMJ Glob Health 2017;2(2):000280. 10.1136/bmjgh-2016-000280 PMC5584490 29242752

[43] Engelgau MM, Karan A, Mahal A (2012). The economic impact of non-communicable diseases on households in India. Glob Health.

[44] Economics DA: Financial impacts of breast cancer in Australia. Sydney: Deliotte. In*.*; 2017.

